# Hair and Blood Trace Elements (Cadmium, Zinc, Chrome, Lead, Iron and Copper) Biomonitoring in the Athletic Horse: The Potential Role of Haematological Parameters as Biomarkers

**DOI:** 10.3390/ani14223206

**Published:** 2024-11-08

**Authors:** Francesca Aragona, Claudia Giannetto, Giuseppe Piccione, Patrizia Licata, Ömer Deniz, Francesco Fazio

**Affiliations:** 1 Department of Veterinary Sciences, University of Messina, Via Palatucci 13, 98168 Messina, Italy; fraragona@unime.it (F.A.); clgiannetto@unime.it (C.G.); gpiccione@unime.it (G.P.); plicata@unime.it (P.L.); 2 Department of Clinical Science and Internal Medicine, Faculty of Veterinary Medicine, Kastamonu University, 37200 Kastamonu, Turkey; odeniz@kastamonu.edu.tr

**Keywords:** blood biomarkers, equine, pollution, trace elements, One Health

## Abstract

The aim of the present study was the evaluation of the bioaccumulation of cadmium (Cd), Zinc (Zn), Chrome (Cr), Lead (Pb), Iron (Fe) and Copper (Cu), in the blood, serum, tail and mane of horses from the industrialized area of Milazzo (Messina, Sicily), to understand the relationships between haematological parameters: Red Blood Cells (RBCs), White Blood Cells (WBCs), Haemoglobin (Hb), Haematocrit (Hct), Mean Corpuscular Volume (MCV), Mean Corpuscular Haemoglobin (MCH), Mean Corpuscular Haemoglobin Concentration (MCHC), Platelets (PLTs) and the concentrations of trace elements. Blood and hair samples from 20 healthy Italian Saddle horses and water, hay and concentrates samples were obtained to determine the haemogram and mineral concentration using a Thermo Scientific iCAP-Q ICP-MS spectrometer. Descriptive analysis showed a higher concentration of Zn, Cr, Pb, Fe, and Cu in the blood and a higher concentration of Cd in the tail than other substrates. A positive correlation was found for Cr (*p* < 0.0001) and Zn (*p* < 0.01) between blood and serum substrates, for Zn (*p* < 0.001) between mane and tail and for Pb (*p* < 0.01) between blood and mane, while a negative correlation was observed for Cr (*p* < 0.01) between blood and tail. Results showed a close relationship between the bioaccumulation of certain trace elements in biological substrates and haematological parameters, which represent useful biomarkers suggesting further studies, given the role of haematological parameters in athletic horses.

## Simple Summary

In the context of biomonitoring, it is important to study animals in an environmental background that could potentially be polluted and to assess all effects on certain haematological parameters as blood biomarkers. Blood and hair are important biological matrices used to assess the bioaccumulation of trace elements in domestic animals. The bioaccumulation of mineral substances in different biological substrates has been evaluated, such as blood, serum, and the mane and tail of horses, as well as in water, hay and concentrate samples, in order to determine the relationship between these trace elements and the substrates. The trace elements analysed showed both positive and negative correlations among biological substrates and direct and indirect haematological parameters. It is evident from the analysis of the results obtained that there is a close relationship between the bioaccumulation of certain trace elements studied in the various equine biological substrates and haematological parameters, which represent, as in the human species, useful biomarkers.

## 1. Introduction

Over the last century, industrialised countries have experienced an exponential increase in anthropogenic activities, increasing levels of environmental pollution [1]. Metals in the environment are considered pollutants, and are essential components of the diet due to their ubiquity and stability characteristics. Improper utilization or dissemination in the environment results in adverse consequences [2,3,4,5,6]. Horses are very useful indicators of environmental contamination due to their proximity to humans, common habitats, and greater organ similarity [7,8]. The horse is a sentinel organism that is subjected to environmental pollution as a grazing animal, directly absorbing all pollutants accumulated in the soil, providing an early warning of the implications that environmental changes may have, for example, for human health [7]. Trace elements such as Zn, Fe and Cu were inherently assimilated by pasture or augmented by hay and concentrates, with their levels contingent upon soil concentrations, fodder maturity, plant type, harvesting conditions, and preservation techniques in grazing animals [7,9,10].

The assessment of metal concentrations in equine tissues and organs gives insights into environmental conditions, and forecasts potential impacts on people and animals. Many biological substrates have been studied in horses, as their levels may reflect acute or chronic exposure to contaminants [11,12,13]. Whole blood, serum, hair, and nails have been used to estimate the level of environmental contaminant concentration in animal biomonitoring studies [7,8,14,15,16]. Whole blood provides insight into short-term exposure to harmful substances; however, primary keratinized structures such as hair, hooves, horns, tails, and manes are preferable, due to their accessibility and the non-invasive, stress-free nature of sampling. These structure, are an inert and chemically homogeneous source of heavy-metal bioaccumulation, indicating a long-term exposure to pollutants [10]. Given their strong reliance on environmental changes, haematological variables may be reliable markers of metal pollution [8,17]. It is intriguing to examine how various contaminants’ bioaccumulation and specific trace elements affect this species’ haematological response to anthropogenic loading [18]. Through blood tests that can be linked to the bioaccumulation of heavy metals, haematological biomarkers are helpful biological indicators that enable us to identify a shift in the body’s homeostasis brought on by exposure to these metals [19]. The haematological profile is a sensitive index for assessing the health condition of domestic animals exposed to ‘metal’ stress [7,16]. In fact, animals exposed to anthropogenic loading of certain heavy metals may initiate a variety of defence mechanisms at the haematological level [20]. Due to the (heavy) metal exposure, the interaction between absorbed metals and blood biomarkers is inevitable. Obtaining an integrated response of the body to the interplay of various toxic effects arising from exposure to a combination of many pollutants is one of the many significant benefits that come with the usage of haematological biomarkers. By anticipating the long-term consequences of the poisonous material, they also enable the body to react to its effects immediately. In recent years, industrial effluents, urban-waste management issues, agricultural activities and expanding urbanization have all posed risks to the biotic and abiotic system. Monitoring pollution activities, their sources and special distribution, and their fate in the environment, is vital in order to conserve species and preserve the regular operation of ecosystems.

Horses who have a large bioaccumulation of some metals may have mild-to-severe toxicosis [2], including peripheral neuropathy, liver illness, gastrointestinal issues, and intermittent colic anaemia, as well as kidney and musculoskeletal diseases [2]. Grazing animals are known to be exposed to Cd deposited on forage or taken up by plants. Iron toxicosis is rarer in horses, as there must be an excess of supplementary administration. Lead is acknowledged as a pernicious worldwide pollutant. This hazardous heavy metal is a multi-media pollutant found in mining and smelting activities, urban areas, and industrial settings. However, the protection of the environment has often been neglected, so monitoring the environments in which domestic animals live is important [2]. There is now a focus on protecting the environment, human and animal health, suggesting a One Health approach, aimed at implementing interdisciplinary actions involving human–animal–environment health [16]. The purpose of the present study was to evaluate the bioaccumulation of some trace elements and their possible harmful effects (Cd, Zn, Cr, Pb, Fe and Cu) released by industrialization in different biological matrices such as blood, serum and hair (tail and mane) and how these trace elements in the body may affect the haematological profile (WBC, HCT, HGB, RBC, PLT, MCHC, MCV, and MCH) of horses. The presence or absence of an effect of these substances on haematological parameters may lead to useful information on the use of these biological parameters as blood biomarkers in the athletic horses.

## 2. Materials and Methods

The Ethics Review Board (Veterinary Department Ethics Committee) of the University of Messina considers that this type of project does not fall under the legislation for the protection of animals used for scientific purposes, national decree-law 113/2013 (2010-63-EU directive). It considers there are no procedures conducted on animals. The blood used in this study was obtained from horses and brought to a laboratory for routine analysis (haemocytometric and biochemical) by a veterinarian, according to good practices. Healthy horses with haematological parameters within physiological reference ranges were considered for the following study [21]. Following clinical tests, the excess blood was obtained by the authors of the manuscript for the following analysis, with the owner’s authorization and written informed consent, as a free donation from the owner for scientific purposes. All animals were considered clinically healthy following a preliminary examination (evaluation of body temperature, heart rate, and respiratory rate) and routine biochemistry, to exclude previous injuries.

Twenty clinically healthy Italian Saddle horses aged between 10 and 15 years old and with a body mass between 435 and 500 kg were used in the present study. They were from the same horse training centre located near the industrialized area of Milazzo (Messina, Sicily 38°00049″ N 15°25018″ E, 80 m above sea level). The experimental group followed a diet based on standard rations of first-cut meadow hay, which was sun-cured, late-cut, and a mixture of cereals and 50% each of oats and barley. The concentrates consisted of the following: 13.2% crude protein, 3.1% crude oils and fats, 11.5% crude cellulose, 8.0% crude ash, 0.2% sodium, 1.2% calcium, and 0.6% phosphorous. The horses in the experimental group received the rations individually three times daily. Water was available ad libitum in both groups. The evaluation of the mineral concentrations present in the hay, concentrate and water for each subject from the experimental group was performed and is reported in Table 1.

Blood samples were collected for all horses in the morning before feeding and physical activity (6:00 a.m.), through the jugular vein using sterilized venojet tubes with ethylenediamine tetra acid (K3EDTA, Terumo Corporation, Tokyo, Japan) anticoagulant in duplicate and without anticoagulant. K3EDTA samples were stored at 4 °C and samples without anticoagulant were centrifuged (1308× *g* × 10 min) for obtaining serum and stored at −20 °C until analysis. Hair samples (tail and mane) were cut using rigid plastic scissors, and pre-cleaned with ethanol before each use, in the part of the neck close to the skin for the mane and tail from where they hung, and each sample was stored in a plastic bag to be analysed. At the same time hay, concentrate, and water were collected in plastic bags and stored at 4 °C. Blood samples with K3EDTA were used (within 4 h) to evaluate trace element concentration and RBC, WBC, Hb, Hct, MCV, MCH, MCHC, and PLT concentration. Blood parameter concentrations were obtained with a species-specific automated haematology analyser (HeCo Vet C, SEAC, Florence, Italy).

The mineral determination of Cd, Zn, Cr, Pb, Fe and Cu was performed on blood, serum, mane and tail by a Thermo Scientific iCAP-Q inductively coupled plasma–mass spectrometry (ICP-MS) spectrometer ASX520 (Cetac Technologies Inc., Omaha, NE, USA) powered by a 27 MHz radiofrequency solid-state generator at 1550 W.

Analyses were performed in triplicate. Linearity, accuracy (% recovery), and sensitivity in term of LOD (limit of detection) and LOQ (limit of quantification) were detected for each element using certified matrices (Whole Blood Metal Control Level 3, Milan, Italy, certified matrix) for blood and hair sample (ERM-DB001), as shown in Table 1. Each procedure followed a specific chemical analysis as described in previous studies for other trace elements in the present biological substrates [7,19].

### Statistical Analysis

The statistical analysis was performed using Prism v. 9.00 (GraphPad Software, San Diego, CA, USA). All data were normally distributed (Kolmogorov–Smirnov test *p* > 0.05). The studied mineral concentration among substrates was analysed by the application of descriptive statistics.

*p* values < 0.05 were considered to be statistically significant. The Pearson correlation coefficient (r) was analysed in order to correlate the concentration of each analysed mineral in blood, serum, tail and mane with the haematological parameters.

## 3. Results

The level of concentration of mineral elements contained in the feed and water was below the limits set by EU regulation no. 744/2012 and EC (European commission 2006) for the studied minerals, as shown in Table 2. The obtained trace elements in horse blood, serum, tail and mane did not show dangerous concentrations, according to the reference values for horses [22].

**Figure 1:**
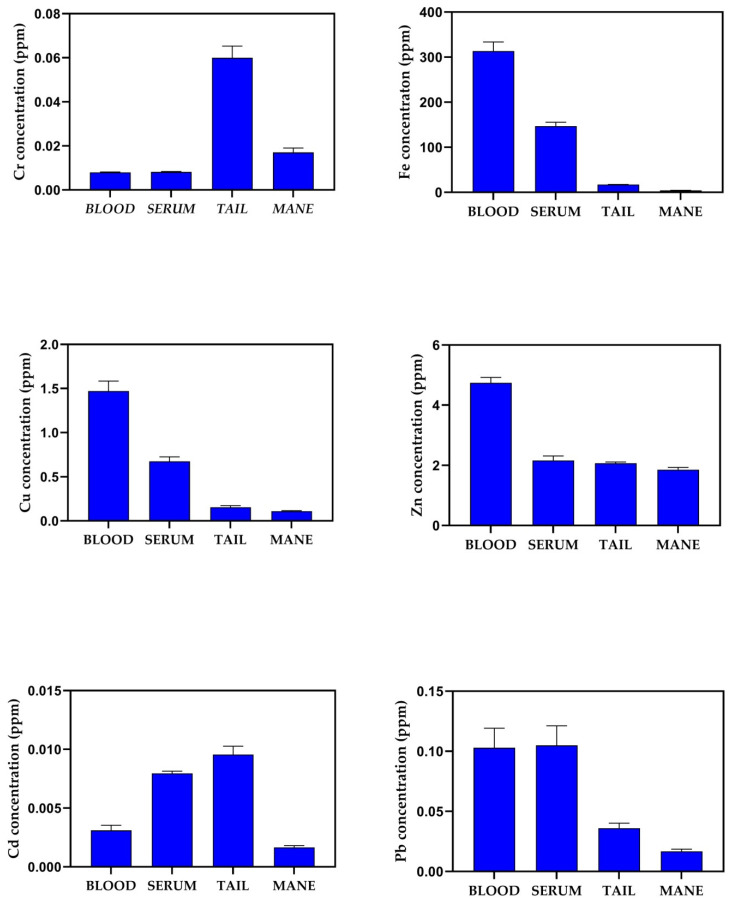
Concentration of Cr, Fe, Cu, Zn, Cd and Pb, in blood, serum, tail and mane of Italian Saddle horses (*n* = 20). Data are represented as mean ± standard error of the mean (SEM).

**Figure 2:**
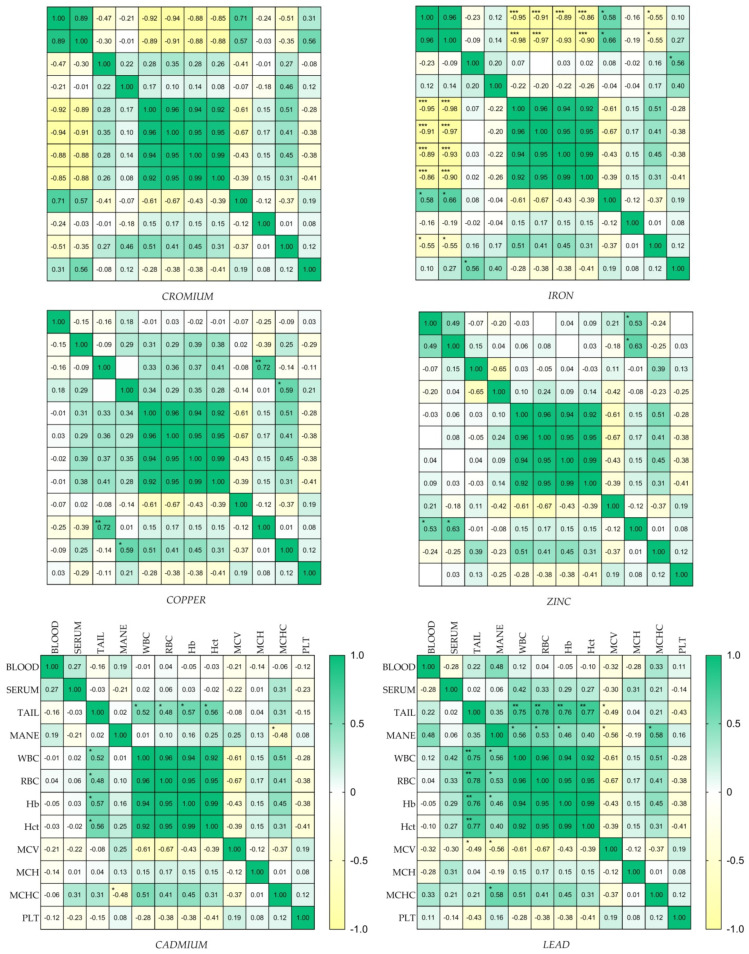
Heat map of correlation analysis (r-values) of Cd, Zn, Cr, Pb, Fe and Cu (ppm) concentration observed among blood, serum, tail and mane and haematological biomarkers (RGB, HCT, HGB, MCV, MCH, MCHC, WBC and PLT) in Italian Saddle horses (*n* = 20) housed near the industrialized area of Milazzo (Sicily, Italy). The symbol * represents *p* < 0.01, ** represents *p* < 0.001, *** represents *p* < 0.0001.

Figure 1 shows Cd, Zn, Cr, Pb, Fe and Cu concentration as mean ± standard error of the mean (SEM) in blood, serum, tail and mane. A higher concentration of Fe, Cu and Zn was observed in blood substrate compared to all other substrates. Higher values of Pb in blood and serum compared to tail and mane were observed, while serum values of Fe and Cu were higher than those for the tail and mane. Higher Cr, Cd and Pb levels were observed in the tail compared to the mane, and higher values of Cr and Cd were observed in the tail substrate compared to all other substrates. The Pearson correlation matrix obtained for each element in the different substrates correlated with haematological biomarkers, as shown in Figure 2.

## 4. Discussion

Trace elements obtained from the blood, serum, tail and mane did not show dangerous concentrations, nor concentrations above the limits observed for those elements in horses, despite the fact that the animals lived close to the industrial area [23,24]. According to the present results, the presence of an industrialized area probably influenced the concentration of minerals obtained in different biological substrates, but it did not lead to evident toxicological effects associated with bioaccumulation in tissues and in the haematological profile of horses [25,26]. Blood and keratinized structures are known to be an essential source of bioaccumulation in humans and animals.

The present results showed different concentration levels of the analysed minerals, depending on the observed substrate.

In particular, a higher concentration of Fe, Cu and Zn was observed in the blood than in other biological substrates, and a higher concentration of Cr, Cd and Pb was observed in the tail than in the mane substrate. Based on the chemical composition of an element, generally, the organism activates numerous biochemical mechanisms to sequester chemical elements and to reduce their potential toxicological impact.

The present results were comparable with other studies which observed higher levels of some mineral content in blood matrices compared to the horsehair samples [11,27,28]. A higher concentration of Cu, Zn and Pb observed in blood compared to other keratinized structures was observed by Aragona et al. in horses living in industrialized and non-industrialized areas [9]. Brummer-Holder [28] observed that blood samples had higher concentrations of some heavy metals (e.g., As and Pb) than hair samples. Kalashnikov et al. [27] observed lower concentrations of toxic metals, including Pb, in the hair of more active horses, as the increased level of sporting activity of horses could be associated with changes in the concentration of certain elements [15,27].

Mineral concentration in blood changes rapidly in response to physiological status; it is responsible for acute short-term changes, and keratinized structures respond more slowly, allowing long-term assays [22,26,27,28].

Human hair is thought to be a significant indication for determining the level of exposure to toxins and pollutants, and it may be utilized to efficiently monitor and prevent pollution in the environment [29].

Our results showed higher values of Cr, Cd and Pb in the tail compared to the mane. The values observed in the tail were comparable with those from hair samples found by Janiszewska and Cieśla [30], (0.039 to 0.997 μg g^−1^) of horses from Poland. Other studies showed that Cd concentration in the horsehair reached values of 0.12 ± 0.12 μg g^−1^ in 24 healthy racehorses in industrialized areas of Japan [26], as well as for horses living in polluted areas in Romania, as reported by Bianu [31]. The tail and mane are both permanent structures that grow continuously, but the tail stands closer to the ground where metals could be absorbed, and it is thinned less frequently than the mane. This information may emphasize the importance of the tail as a bioaccumulation matrix that could give us a better idea of the chronic exposure to some contaminants in the environment.

Haematological parameters, evaluated in horses, could be used as blood biomarkers in response to the bioaccumulation of heavy metals in different biological matrices (blood, serum, tail and mane). It was observed that the concentration of Cr in the blood substrate could lead to a reduction of WBCs, RBCs, Hct, Hb and MCHC, as well as the concentration of Cr and Fe in serum. Furthermore, the Cd and Pb concentration in the tail could positively influence the number and amount of WBCs, RBCs and Hb, just as the Pb concentration observed in the mane could influence the number and amount of WBsC, RBCs, Hb and MCHC. Thus, it emerges, in contrast to previous studies in sheep, and in horses in which no correlations were observed between metal concentrations in biological substrates and haematological parameters [9,16], that there is a relationship between blood parameters and the concentration of the metals analysed, in agreement with Fazio et al. [11]. The use of haematological parameters as biomarkers can be useful to highlight a bioaccumulation or mineral deficiency in the horse’s organism. Deficiencies or bioaccumulation of certain elements such as Cd, Zn, Cr, Pb, Fe and Cu, based on the present results, could be used in combination with the number of blood cells for a more precise clinical analysis and biomonitoring. Thus, a large number of subjects should be advisable in further studies, as well as the same experiment conducted simultaneously, even in a healthy environment, to address the limitation of the present study.

## 5. Conclusions

The present study emphasized the effect of bioaccumulation of certain trace elements in different biological substrates and the positive and negative influence of these concentrations on haematological profile of horses. The impact of the geographical polluted area did not show any particular negative signs in horses. The fact that long-term exposure, in addition to being harmful to the environment can go on to affect animals and human health, cannot be excluded. It may open the door to future studies considering the One Health approach for the prevention of risk factors for humans, animals, and the environment. Moreover, it would be important to promote good management practices and welfare for the athletic horses that inhabit areas with high anthropogenic impact, considering the evaluation of the haematological profile that is crucial for good performance.

## Figures and Tables

**Table 1:** Mean ± standard deviation (SD) validation parameters of ICP-MS and DMA-80 methods were performed in terms of linearity, limit of detection (LOD), limit of quantification (LOQ), and accuracy (% of recovery) for each trace element.

	LOD (μg/kg)	LOQ (μg/kg)	R^2^	Recovery
CADMIUM	0.001 ± 0.000	0.003 ± 0.000	0.999	101.25 ± 0.50
ZINC	0.057 ± 0.016	0.18 ± 0.04	0.999	98.85 ± 0.68
CROMIUM	0.001 ± 0.000	0.003 ± 0.000	0.999	98.22 ± 0.56
LEAD	0.001 ± 0.000	0.003 ± 0.000	0.999	100.80 ± 0.48
IRON	0.010 ± 0.004	0.045 ± 0.015	0.999	97.87 ± 0.71
COPPER	0.015 ± 0.007	0.050 ± 0.013	0.970	97.00 ± 0.50

**Table 2:** Metal concentration ± standard deviation (SD) of horse-fed water, hay and concentrate values (mg/L- mg/Kg of dry matter). The symbol * indicates the concentration below the limit of quantification (LOQ).

	WATER	HAY	CONCENTRATE
CADMIUM	*	*	*
ZINC	0.004 ± 0.0001	3.02 ± 0.2	5.73 ± 0.01
CROMIUM	0.005 ± 0.0001	0.05 ± 0.003	0.01 ± 0.002
LEAD	0.008 ± 0.001	0.023 ± 0.04	153 ± 2.50
IRON	0.42 ± 0.001	0.4 ± 0.02	153 ± 2.50
COPPER	0.008 ± 0.001	0.28 ± 0.02	0.1 ± 0.02

## Data Availability

The raw data supporting the conclusions of this article will be made available by the authors on request.
